# HOTTIP Functions as a Key Candidate Biomarker in Head and Neck Squamous Cell Carcinoma by Integrated Bioinformatic Analysis

**DOI:** 10.1155/2019/5450617

**Published:** 2019-03-26

**Authors:** Xiteng Yin, Weidong Yang, Junqi Xie, Zheng Wei, Chuanchao Tang, Chuanhui Song, Yufeng Wang, Yu Cai, Wenguang Xu, Wei Han

**Affiliations:** ^1^Department of Oral and Maxillofacial Surgery, Nanjing Stomatological Hospital, Medical School of Nanjing University, Nanjing, China; ^2^Central Laboratory of Stomatology, Nanjing Stomatological Hospital, Medical School of Nanjing University, Nanjing, China; ^3^Department of Endodontics, Nanjing Stomatological Hospital, Medical School of Nanjing University, Nanjing, China

## Abstract

**Background:**

Accumulating evidence has demonstrated the pivotal role of long noncoding RNAs (lncRNAs) in competing endogenous RNA (ceRNA) networks for predicting survival and evaluating prognosis in cancer patients. However, the pathogenesis of head and neck squamous cell carcinoma (HNSCC) remains unclear, and prognostic biomarkers for HNSCC are still lacking.

**Methods:**

A total of 546 RNA sequencing profiles of HNSCC patients with clinical outcome data were obtained from the Cancer Genome Atlas (TCGA) database, providing a large sample of RNA sequencing data. From these, 71 Long noncoding RNAs lncRNAs, 8 microRNAs (miRNAs), and 16 messenger RNAs (mRNAs) were identified to construct a HNSCC-specific ceRNA network (fold change >2, P < 0.05). Univariate and multivariate Cox proportional regression models were used to assess independent indicators of prognosis. Then the expression of lncRNAs harboring prognostic value was validated in human HNSCC cell lines and tumor samples from our cohort and another two datasets from GEO (Gene Expression Omnibus) databases.

**Results:**

As a result, a 3-mRNA signature and 6-lncRNA signature were identified. The six-lncRNA signature exhibited the highest prognostic value. Notably, in the six lncRNAs, HOTTIP showed the greatest prognostic value and was significantly correlated with clinical stage and histological grade of HNSCC patients. Furthermore, it was proved that HOTTIP was upregulated in HNSCC cell lines and cancerous tissues compared with corresponding normal cell lines and normal tissues. Functional assessment analysis revealed that HOTTIP might play a key role in the oncogenesis and progression of HNSCC.

**Conclusion:**

The present study deepened our understanding of the ceRNA-related regulatory mechanism in the pathogenesis of HNSCC and identified candidate prognostic biomarkers for clinical outcome prediction in HNSCC. HOTTIP may function as a key candidate biomarker in HNSCC and serve as a prognostic marker for HNSCC patients.

## 1. Introduction

Head and neck cancer ranks as the sixth leading cancer worldwide, among which head and neck squamous cell carcinoma (HNSCC) is the most common subtype, accounting for more than 90% of cases [1]. Despite technological advances in early detection and intervention and escalation of treatment modalities, clinical outcomes and long-term survival for patients with HNSCC have not improved significantly, with the 5-year survival rate remaining at 50%–60% [2]. The American Joint Committee on Cancer staging system is used to classify and stage HNSCC, which is essential for clinicians to assign appropriate treatments and evaluate the prognoses of HNSCC patients [3]. However, it has been observed in clinical practice that some patients with early stage cancers have an unexpectedly worse prognosis than those with advanced stage cancers. It is therefore necessary to identify potential biomarkers, for use in addition to clinical and pathological factors, for more precise evaluations of prognosis and to gain insights into the detailed molecular mechanisms of HNSCC.

Long noncoding RNAs (lncRNAs) are defined as RNAs of longer than 200 nucleotides that are not translated into protein because they have few or no open reading frames [4]. A growing body of evidence indicates that lncRNAs play a critical role in the initiation and progression of multiple cancers [5]. However, their functions in HNSCC have not been fully investigated. Accumulating recent studies have suggested that interactions between lncRNAs and microRNAs (miRNAs) occur in HNSCC, and some of their downstream target genes have been confirmed to correlate with tumor initiation and progression. In 2011, Salmena et al. proposed a competing endogenous RNA (ceRNA) hypothesis that depicts a complicated posttranscriptional regulatory network, in which lncRNAs act as ‘sponges', soaking up miRNAs by binding to miRNA response elements, thereby unsuppressing the target genes of the respective miRNAs [6]. Various lncRNA and miRNA interactions with significant functions have been confirmed in HNSCC, suggesting potential roles as diagnostic and prognostic markers for this condition [7–9]. However, the prognostic value of lncRNA-associated ceRNA regulatory networks in HNSCC remains unclear. Some lncRNAs have been shown to predict long-term outcomes for HNSCC patients [10, 11], but the conclusions from these studies remain inconsistent, possibly owing to the relatively small sample sizes.

The Cancer Genome Atlas (TCGA) project, launched in 2005, has generated comprehensive, multidimensional maps of the key genomic changes across 33 types of cancer. It uses genome sequencing and high-throughput genome analysis techniques and has collected relevant clinical data from 11 000 patients. With such an enormous amount of data, TCGA provides a rich resource for data mining and identifying potential biomarkers [12].

In the present study, the integrated RNA expression profiles of HNSCC patients along with their clinical outcomes were obtained from the TCGA database to construct a lncRNA–miRNA–mRNA ceRNA network for HNSCC. Through an integrated and comprehensive analysis of lncRNA expression patterns in the ceRNA network, we attempted to elucidate the interactions and potential crosstalk between differentially expressed lncRNAs, miRNAs, and mRNAs. In addition, survival analyses were performed to identify the relative lncRNA, mRNA, and microRNA prognostic signatures involved in the ceRNA network as new candidate prognosticators to predict the overall survival of HNSCC patients.

## 2. Materials and Methods

### 2.1. The Cancer Genome Atlas Dataset and Patient Information

RNA sequencing and miRNA sequencing data were obtained from the TCGA data portal (https://portal.gdc.cancer.gov/). Data were collected relating to HNSCC tissue samples and nontumorous tissue samples from adjacent normal tissue collected up to February, 2018. Given that the data were obtained from the TCGA database, additional approval from the Ethics Committee was not needed. Data processing procedures met the requirements of the data access policies and the National Institutes of Health (NIH) TCGA human subject protection.

### 2.2. Identification of Differentially Expressed RNAs in Head and Neck Squamous Cell Carcinoma Samples

The profiling of human RNAs, including mRNA, lncRNA, and miRNA in HNSCC samples was based on RNASeqV2 and Illumina HiSeq 2000 miRNA sequencing platforms. Level 3 RNA expression data of HNSCC patients were obtained from the TCGA data portal and normalized by TCGA program. To screen out the differentially expressed RNAs, samples were divided into tumor tissues versus normal tissues. Differentially expressed RNAs were identified by fold change and adjusted P values. Fold changes of >2 and <0.5 and false discovery rate, namely, the adjusted P < 0.05, were determined as statistically significant. The detailed steps of identification of differentially expressed RNAs were in accordance with studies previously reported by Sui et al. [13].

### 2.3. Prediction of lncRNAs and mRNAs Targeted by miRNAs

Relevant information on miRNAs and lncRNAs was integrated according to miRcode (http://www.mircode.org/) to predict lncRNA–miRNA interactions. The miRTarBase (http://mirtarbase.mbc.nctu.edu.tw, release 6.0), miRDB (http://www.mirdb.org/), and TargetScan (http://www.targetscan.org/vert_71/) were utilized to search for potential target genes of the identified miRNAs.

### 2.4. Construction of the ceRNA Network and Functional Assessment

According to the predicted relationships between lncRNA, mRNA, and miRNA in HNSCC, the ceRNA network for HNSCC was established. Cytoscape v3.0 was utilized to construct and visualize the ceRNA network. To identify the underlying pathways and biological processes of differentially expressed genes in the ceRNA network, the Database for Annotation, Visualization, and Integrated Discovery (DAVID) (http://david.abcc.ncifcrf.gov/) was used for functional enrichment analysis. The KEGG (Kyoto Encyclopedia of Genes and Genomes) pathways and GO (Gene Ontology) terms were obtained with the significance level of P < 0.05 and the enrichment score of >1.5 [13].

### 2.5. Identification of the Head and Neck Squamous Cell Carcinoma-Specific Prognostic Signatures

In order to determine novel prognostic biomarkers involved in the ceRNA network, HNSCC-specific mRNAs, lncRNAs, and miRNAs were extracted from the ceRNA network, and the expression level of each RNA was log^2^ transformed for subsequent analysis. The univariate Cox proportional hazards regression model with a significance level set at 0.05 was employed to identify HNSCC-specific RNAs associated with overall survival. Kaplan–Meier curves and log-rank methods (Mantel–Haenszel test) were further used to visualize the survival distributions in different groups and compared using R package “survival”.

In accordance with further multivariate Cox proportional hazards regression analysis, we constructed HNSCC-specific mRNA, lncRNA, and miRNA prognostic models. The prognostic risk score for predicting overall survival was calculated: risk score = exp_RNA1_ × *β*_RNA1_ + exp_RNA2_ × *β*_RNA2_ + …exp_RNAN_ × *β*_RNAn_ (where exp is the expression level and *β* is the regression coefficient derived from the multivariate Cox regression model). Using the median risk score as the cutoff point, HNSCC patients were stratified into high-score and low-score groups as previously reported [13, 14].

A 1-year, 3-year, 5-year, and 10-year time-dependent receiver operating characteristic (ROC) curve analysis were performed using “survival-ROC” package in R software to assess the predictive value of the risk score for time-dependent outcomes [13, 14]. The ROC curve was used to evaluate the sensitivity and specificity of HNSCC-specific signatures for predicting overall survival of HNSCC patients.

### 2.6. Cell Culture and Patients' Samples

HNSCC cell lines (Detroit 562, CAL27, JHU011, HN4, HSC3, SCC4) and human immortalized oral epithelial cell (HIOEC) were obtained from the Cell Bank of the Chinese Academy of Sciences (Shanghai, China). Cells were cultured in a monolayer in DMEM supplemented with 10% FBS, 100 *μ*g/mL streptomycin, and 100 U/mL penicillin in a humidified incubator (5% CO2 /20% O2) at 37°C. Confluent cells were trypsinized with 0.05% trypsin containing 0.02% ethylenediaminetetraacetic acid. Tumor tissues from 3 HNSCC patients were obtained from the biobank of Nanjing Stomatological Hospital. Institutional review board approval was gained for this study from Nanjing Stomatological Hospital Ethics Committee, approval number: 2018NL-010(KS).

### 2.7. Microarray Data Information from Gene Expression Omnibus (GEO) Database

GEO is a public functional genomics data repository containing array- and sequence-based data, from which the gene expression profiles of GSE59652 and GSE89657 of head and neck cancer and normal tissue were obtained. These two series, GSE59652 and GSE89657, included 7 HNSCC tissues and 7 normal tissues, and 6 HNSCC tissues and 6 normal tissues, respectively. GSE59652 series was based on GPL13825 Arraystar Human LncRNA microarray V2.0 (Agilent-033010 Feature Number version) and GSE89657 was based on GPL16956 Agilent-045997 Arraystar human lncRNA microarray V3 (Probe Name Version).

### 2.8. Quantitative Real-Time PCR Assay

Total RNA was extracted from HNSCC cell lines and tumor samples using Trizol Reagent (Invitrogen, Invitrogen, Cat. No. 15596-026, USA), and total RNA was reverse-transcribed with the PrimeScript RT-PCR Kit (Takara Bio, Code No. RR036A, Japan). RT-PCR was performed using Premix Ex Taq reagent (Takara Bio, Otsu, Japan). PCR was performed on ViiA™ 7 (Thermo Fisher Scientific) using the ChamQ Universal SYBR qPCR Master Mix (Vazyme Biotech, Nanjing, China) according to the manufacturer's protocol. The primer sequences used were as follows: LINC00052: forward 5'-AGCTCTCTCACCATGCGATT-3', reverse 5'-TGTTTGCAGACTGTAGGGCT-3', ZFY-AS1: forward 5'-TTGGAACCCATTTCTTCAGG, reverse 5'- ATTCACTCCCTCTGCTTCCA-3', ABCA9-AS: forward 5'-AAAGAGGCTGCCATCGGTAT-3', reverse 5'-TGTGCTGGGCTCTTACAAGT-3', MIAT: forward 5'-TTCTAATGGTGGGAGGGCAG-3', reverse 5'- CTCCCCTGAAAATGGACCCT-3', HOTTIP: forward 5'-AGAAAGGGTCTCAGCTCCAC-3', reverse 5'-AGGCAGGGCTGTACTCAAAT-3', LINC00460: forward 5'-TGCACACTTCTCGGCTAAGA-3', reverse 5'-TTCCCACGCTCAGTCTTTCT-3', HOXA10: forward 5'-GGTACGGACAGACAAGTGAAAATCTT-3', reverse 5'-GGAAGTGAAAAAACCGCGTCGCCTGG-3'; *β*-actin: forward 5'-CTCTTCCAGCCTTCCTTCCT-3', reverse 5'-AGCACTGTGTTGGCGTACAG. The ubiquitously expressed *β*-actin mRNA fragment (190 bp) was amplified as an internal control for RNA quality. For microRNA miR195-5p, it was reverse-transcribed with the riboSCRIPT Reverse Transcription Kit (100T) (Ribobio, C11027-2, China). The primer sequence of miR195-5p was acquired from Bulge-Loop has-miR195-195-5p Primer Set, 100T (Ribobio, MQPS0000758-1-100, China). All data were acquired from at least 3 independent experiments.

### 2.9. Statistical Analysis

The statistical significance of HOTTIP expression between the groups of different clinical features was analyzed using Kolmogorov–Smirnov tests. The correlation analysis of HOTTIP with target genes' expression was performed by Pearson test, and correlation plots were generated by R packages “corrplot”. Values of P < 0.05 were considered to indicate a statistically different result.

## 3. Results

### 3.1. Identification of Differentially Expressed mRNAs, lncRNAs, and miRNAs

RNA and miRNA sequence data from 546 tissue samples (44 normal tissue samples and 502 HNSCC tissue samples from a total of 546 patients) were acquired, and the corresponding clinical data were obtained from the TCGA database. By comparing the expression of RNAs between HNSCC tissue samples and adjacent normal tissue samples, we identified some differentially expressed mRNAs, lncRNAs, and miRNAs (fold change ≥2, adjusted P < 0.05) (Supplementary Figure 1). A total of 873 upregulated and 1128 downregulated genes were identified from mRNA analysis (Supplementary File 1). From the lncRNAs, 1052 genes were found to be differentially regulated, among which 766 were upregulated and 286 were downregulated (Supplementary File 2). Among the miRNAs, 44 genes were upregulated and 38 were downregulated (Supplementary File 3). In addition, with a cutoff P value of < 0.05, we identified significantly enriched GO terms and signal pathways via functional assessment of the differentially expressed mRNAs (Supplementary Figure 2). The top 5 significantly enriched signal pathways are “protein digestion and absorption”, “salivary secretion”, “calcium signaling pathway”, “ECM-receptor interaction”, and “PPAR signaling pathway”.

### 3.2. The ceRNA Network

**Figure 1:**
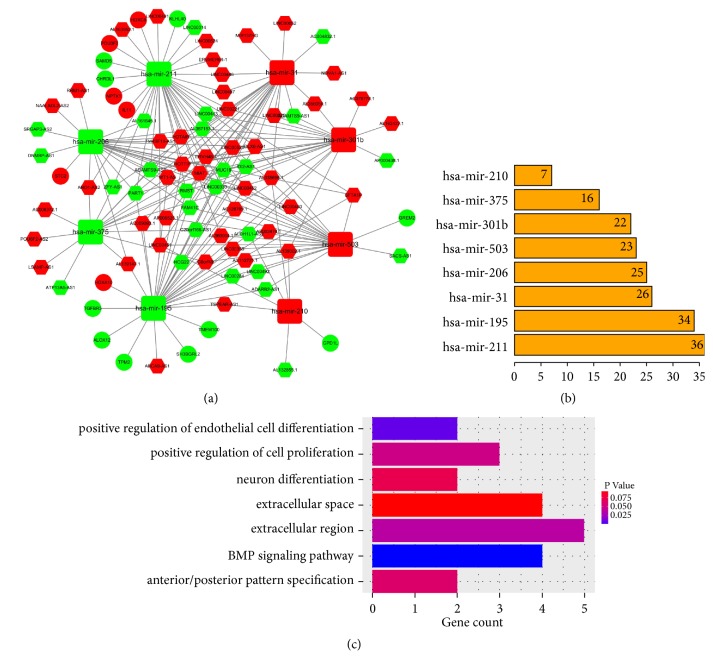
*The HNSCC-specific lncRNA–miRNA–mRNA ceRNA network.* (a) Construction of the HNSCC-specific lncRNA–miRNA–mRNA ceRNA network. Green circles: downregulated mRNAs; red circles: upregulated mRNAs; green hexagons: downregulated lncRNAs; red hexagons: upregulated lncRNAs; green squares: downregulated miRNAs; red squares: upregulated mRNAs. Grey lines denote lncRNA–miRNA and miRNA–target gene interactions. (b) Nodes with the highest degrees of connectivity in the ceRNA network. (c) Enriched pathways and functions of target genes in the ceRNA network by GO analysis.

Database searches predicted 173 miRNA–lncRNA pairs, in which 8 differentially expressed miRNAs interacted with 71 differentially expressed lncRNAs (Supplementary File 4). The target mRNAs of the 8 differentially expressed miRNAs corresponded to 395 target genes. Of the mRNAs of these target genes, 16 had been identified as being differentially expressed. The final HNSCC-specific ceRNA network therefore comprised 8 miRNAs (4 downregulated and 4 upregulated), 16 mRNAs (10 downregulated and 6 upregulated), and 71 lncRNAs (26 downregulated and 45 upregulated) (Supplementary Table 1). It was constructed with 95 nodes and 189 edges (Figure 1). Of the 95 nodes, 5 miRNAs (hsa-mir-211, hsa-mir-195, hsa-mir-31, hsa-mir-206, and hsa-mir-503) were hub nodes that were highly connected (Supplementary Figure 2), which indicated that these miRNAs might have central roles in connecting lncRNAs and mRNAs. Furthermore, we performed GO analysis of the 16 mRNAs involved in the ceRNA network to identify possible signal pathways that may be indirectly regulated by lncRNAs, including “positive regulation of endothelial cell differentiation”, “positive regulation of cell proliferation”, “extracellular region”, and “BMP signaling pathway” (Supplementary Figure 3).

### 3.3. mRNA, lncRNA, and miRNA Prognostic Signatures

With the establishment of HNSCC-specific ceRNA network, we aimed to identify novel prognostic biomarkers involved in the ceRNA network for HNSCC. From the univariate Cox proportional hazards regression analysis, 3 miRNAs, 15 lncRNAs, and 1 miRNA were identified as being significantly related to overall survival in HNSCC patients (P < 0.05) (Supplementary Table 2). Multivariate Cox proportional hazards regression analysis showed that 3 mRNAs, 6 lncRNAs, and 1 miRNA were strongly prognostic, and these were incorporated into a multifactor prognostic model (Supplementary Table 3). Among the 3 mRNAs included in the multivariate model, only* STC2* acted as a significant independent prognosticator for overall survival in HNSCC (P < 0.001), which suggests that* STC2* might play a vital role in the progression of HNSCC. Five of the six lncRNAs were significantly prognostic (P < 0.05), with* HOTTIP* having the strongest prognostic value (P < 0.001). Kaplan–Meier curves and log-rank tests were performed to visualize the prognostic value of mRNAs, lncRNAs, and miRNAs involved in the ceRNA network, and 1 mRNA, 13 lncRNAs, and 1 miRNA were validated to be closely connected with overall survival of HNSCC patients (Supplementary Figure 3).

**Figure 2:**
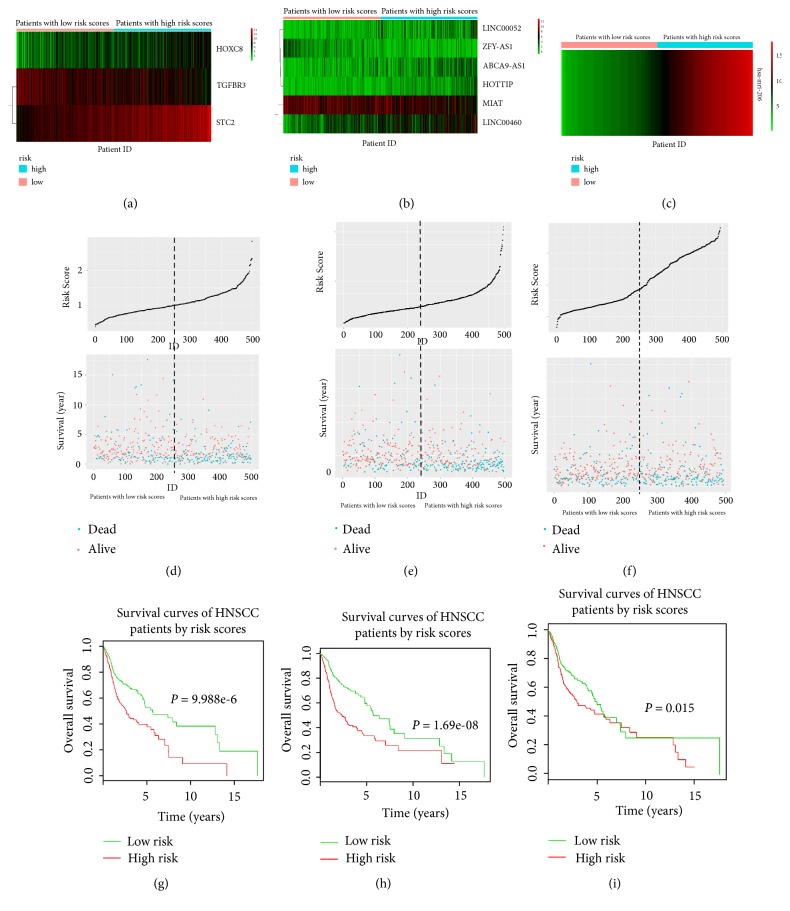
*Risk score analysis of the differentially expressed mRNA, lncRNA, and miRNA signature of HNSCC.* (a-c) Heatmaps of mRNA, lncRNA, and miRNA expression profiles of HNSCC patients ranked according to the predicative risk score values for each signature. (d-f) Risk score and survival status distribution of each case for each signature. (g-i) KM survival curves for overall survival of HNSCC patients, respectively, with high- and low-risk score.

Based on the multivariate Cox proportional hazards regression analysis, we developed the mRNA, lncRNA, and miRNA prognostic model for HNSCC, respectively. The gene expression level was as the log2 reads per million of total aligned RNA reads. The prognostic score was calculated as follows: for mRNAs, risk score=Exp_TGFBR3_*∗*(-0.09) +Exp_STC2_*∗*0.1773 +Exp_HOXC8_*∗* 0.0616; for lncRNAs, risk score=Exp_LINC00052_*∗*0.06305+Exp_ZFY-AS1_*∗*(-0.11701) +Exp_ABCA9-AS_1*∗*0.08314+ Exp_MIAT_*∗*(-0.09672) +Exp_HOTTIP_*∗*0.15679 +Exp_LINC00460_*∗* 0.0696; for miRNA, risk score=Exp_hsa-mir-206_*∗* 0.03602. Then, HNSCC patients were stratified into low-risk and high-risk groups based on prognostic risk score of the three signatures (Figure 2). Moreover, through depicting the Kaplan–Meier curves of low-risk group and high-risk group, it was confirmed that HNSCC patients in low-risk group had significantly better prognosis than those in high-risk group (P<0.05). Notably, among the three prognostic signatures, the six-lncRNA signature exhibited the highest prognostic value (P<0.001).

The ROC analysis suggested that the six-lncRNA signature had greater prognostic performance than the other two signatures in terms of area under the curve (AUC) values (Supplementary Figure 4). It is noteworthy that the six-lncRNA signature exhibited the greatest prognostic capacity for predicting 3-year survival in HNSCC with an AUC of 0.708.

### 3.4. HOTTIP Was Upregulated in HNSCC and Correlated with Tumor Progression

**Figure 3:**
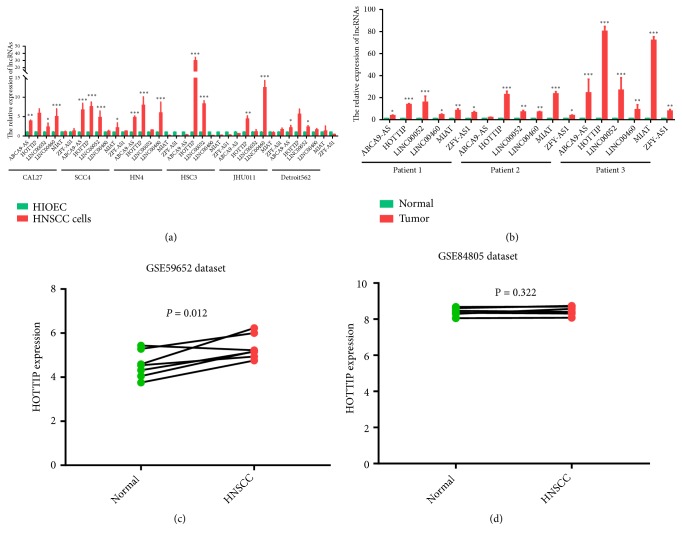
*The expression of the six lncRNAs in HNSCC.* (a) HOTTIP expression was detected in HNSCC cell lines (CAL27, SCC4, HN4, HSC3, JHU011, and Detroit 56) and HIOEC cells by qPCR assays. (b) HOTTIP expression was detected in HNSCC samples and normal tissues by qPCR assays. (c-d) HOTTIP expression in HNSCC samples and paired normal tissues in GSE59652 and GSE89657 series. *∗*p<0.05, *∗∗*p<0.01, *∗∗∗*p<0.001.

Subsequently, the transcriptional expression of the six-lncRNA signature was further validated in HNSCC cell lines and HNSCC patients. The expression of six lncRNAs was verified in 6 HNSCC cells and 3 paired HNSCC and adjacent normal tissues from patients. It was striking that HOTTIP was upregulated in most of HNSCC cells and cancerous tissues in comparison with HIOEC cells and normal tissues, respectively (Figures 3(a) and 3(b)). In addition, the HOTTIP expression was validated in another two datasets from GEO database. The results showed that HOTTIP expression of HNSCC samples was higher than that in normal tissues in GSE59652 series. However, in GSE89657 series, HOTTIP expression did not show significantly differential expression between HNSCC samples and normal tissues (Figures 3(c) and 3(d)), which may be caused by batch effects or different chip platforms employed in the assay.

**Figure 4:**
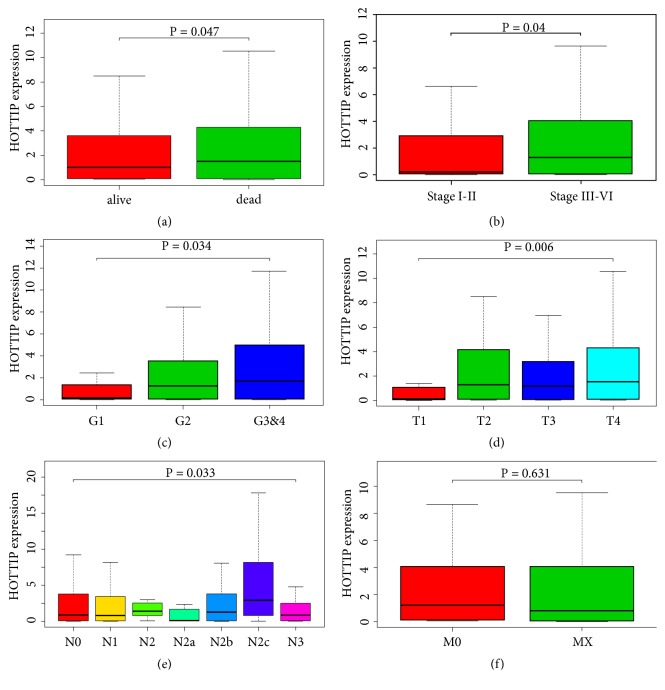
*The HOTTIP expression levels in HNSCC tissues stratified by major clinical features including survival status* (a), clinical stage (b), histological grade (c), T stage (d), N stage (e), and M stage (f).

Given that HOTTIP showed the strongest prognostic value among the six lncRNAs and given its upregulated expression in HNSCC cell lines and patients' samples, it is suggested that HOTTIP might play a key role in pathogenesis and progression in HNSCC. Thus, the relationships of HOTTIP expression and major clinical parameters including clinical stage, histological grade, survival status, T stage, N stage, and M stage were further analyzed. It was shown that HOTTIP expression was closely associated with clinical stage, histological grade, and survival status. HOTTIP was highly expressed in stage III-IV and histological grade 3-4 subgroups compared with stage I-II and histological grade 1-2 subgroups, respectively (P<0.05) (Figure 4).

### 3.5. Target Prediction and Functional Enrichment of HOTTIP in HNSCC

**Figure 5:**
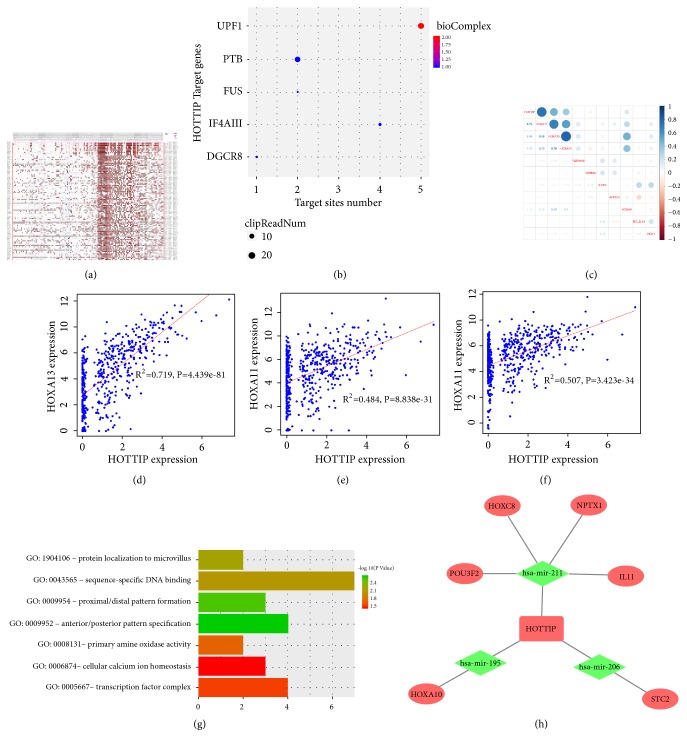
*Target gene prediction of HOTTIP and functional assessment.* (a) Target genes of HOTTIP predicted by Multi-Experiment Matrix; (b) target genes of HOTTIP predicted by starBase; (c) correlation plots of HOTTIP expression and top 10 target genes in TCGA HNSCC cohort; (d)-(f) scatter plots of the correlation analysis of HOTTIP expression with HOXA13, HOXA10, and HOXA11; (g) enriched GO terms by target genes of HOTTIP; (h) HOTTIP-miRNA-mRNA network.

Then, the target genes of HOTTIP were predicted by Multi-Experiment Matrix (MEM) and starBase. A total of 78 target genes from MEM and five genes from starBase were acquired (Figures 5(a) and 5(b)). To confirm the relationship of HOTTIP and its target genes, we performed correlation analysis between HOTTIP and top ten target genes obtained from MEM in TCGA HNSCC patients (Figure 5(c)). As a result, HOXA13, HOXA11, and HOXA10 were shown to significantly correlate with HOTTIP (r=0.719,0.484 and 0.507, respectively; P<0.05).

To explore the biological function of potential target genes of HOTTIP, GO enrichment and KEGG pathway analyses were performed. The GO analysis revealed that seven GO terms were enriched (Figure 5(g)). The GO terms enriched by target genes were mainly related to tumorigenic function such as: protein localization to microvillus, sequence-specific DNA binding, and transcription factor complex.

**Figure 6:**
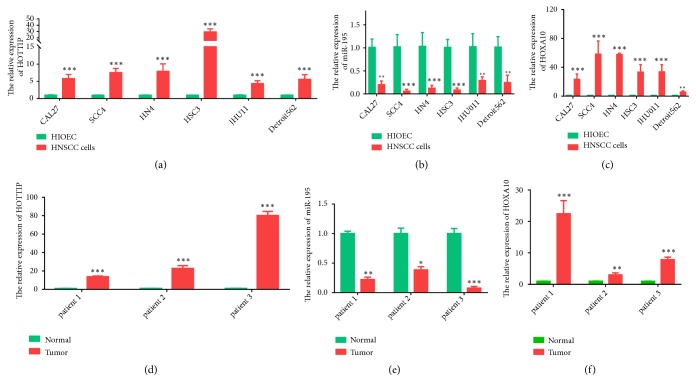
*Expression pattern of HOTTIP-miR195-HOXA10 in HNSCC.* (a)-(c) The expression of HOTTIP, miR195, and HOXA10 in HNSCC cell lines compared with HIOEC cells. (d)-(f) The expression of HOTTIP, miR195, and HOXA10 in HNSCC samples compared with normal tissues.

Finally, based on the target prediction genes prediction by Multi-Experiment Matrix (MEM) and starBase databases, a combined ceRNA network focusing on HOTTIP was established, including 6 mRNAs and 3 microRNAs (Figure 5(h)). To provide evidence for the possible ceRNA regulatory mechanism of HOTTIP, we performed a pilot study on the HOTTIP-miRNA-mRNA network by detecting the expression pattern of HOTTIP-miR195-5p-HOXA10 in HNSCC cell lines and tumor samples in comparison to normal cell lines and normal tissues. Results showed that HOTTIP along with HOXA10 was upregulated in HNSCC cell lines and tumor samples, while miR195 was downregulated in HNSCC cell lines and tumor samples (Figure 6), which indicated that HOTTIP might increase HOXA10 expression through downregulating miR195.

### 3.6. Functional Role of HOTTIP in the Progression of HNSCC

**Figure 7:**
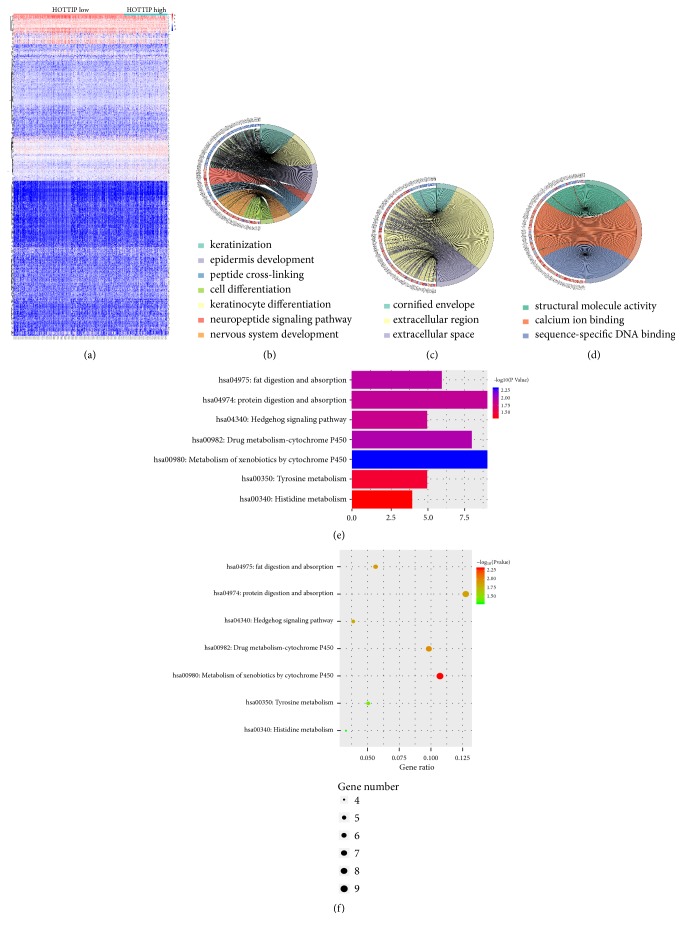
*Potential functional pathways that HOTTIP may be involved in for HNSCC*. (a) Heatmap of the differentially expressed genes (DEGs) between HOTTIP high subgroups and HOTTIP low groups in TCGA HNSCC cohort; (b-d) enriched GO biological process (b), cellular component (c), and molecular function (d) terms by DEGs; (e-f) enriched KEGG pathways by DEGs.

To further investigate the potential role of HOTTIP in HNSCC, the patients in the TCGA cohort were divided into two groups according to geometrical mean of HOTTIP expression. Using |logFC| ≥1 and p<0.05 as the cutoff criteria, a total of 891 differentially expressed genes (DEGs) were identified including 723 upregulated genes and 168 downregulated genes in HOTTIP high expressed HNSCC tissues compared to HOTTIP low expressed HNSCC tissues (Figure 7(a)). We next performed GO enrichment and KEGG pathway analyses to elucidate the biological function of DEGs and role of HOTTIP in HNSCC. The enriched GO terms were shown in Figures 7(b)–7(d), including seven biological process terms, three cellular component terms, and three molecular function terms. This analysis revealed an overrepresentation of the DEGs involved in the critical pathway linked to tumor progression function such as keratinocyte differentiation, cell differentiation, and structural molecule activity. The KEGG analysis showed that the DEGs were mainly enriched in protein digestion and absorption, drug metabolism by cytochrome P450, Hedgehog signaling pathway, and tyrosine metabolism (Figures 7(e) and 7(f)). These indicate a potentially important functional role of HOTTIP in the progression of HNSCC.

Taken together, these exploratory analyses suggest that HOTTIP played an important role in the oncogenesis and progression of HNSCC, yet the underlying mechanism warrants follow-up research.

## 4. Discussion

HNSCC develops as a result of a series of genetic and epigenetic alterations in tumor suppressor genes and oncogenes, resulting in the initiation and progression of the disease. Both the complex mechanisms of HNSCC and the lack of effective and practical biomarkers lead to dismal prognosis [15]. Therefore, identifying novel molecular biomarkers will improve diagnosis and prediction of prognosis in HNSCC. Tremendous advances in molecular biology have contributed to improved understanding at the molecular level of the pathogenesis of this disease, and consequently diagnosis and prognosis have largely improved in recent years. Numerous molecular signatures for HNSCC prognosis have been discovered [16–18].

There is a growing body of evidence showing that lncRNAs may play important roles in the oncogenesis and progression of HNSCC. Some of the most widely studied lncRNAs both* in vivo* and* in vitro* in HNSCC are HOTAIR, H19, MALAT-1, and UCA1 [11, 19–21]. They are involved in various key cellular processes such as proliferation, apoptosis, migration, and invasion. For example, HOTAIR was shown to be overexpressed in HNSCC and closely connected with poor prognosis of HSNCC patients [22]. Targeting HOTAIR could induce apoptosis and inhibit tumor growth in HNSCC both* in vitro* and* in vivo* [7].

Accumulating studies have demonstrated the involvement of lncRNAs in ceRNA networks, whereby lncRNAs can act as microRNA decoys to regulate gene expression. Various ceRNA network models have been established in different types of malignancies, such as hepatocellular carcinoma, gastric carcinoma, and lung carcinoma [13, 23, 24]. In the present study, a putative ceRNA network was constructed by integrating data on lncRNA, miRNA, and mRNA expression from a large sample of HNSCC patients from the TCGA database in accordance with specific criteria. More importantly, we identified prognostic signatures among RNAs involved in the ceRNA network and developed risk scores to predicted overall survival in HNSCC patients.

To date, some lncRNA-related prognostic signatures in HNSCC have been identified [10, 25–27]. It was noteworthy that a three-lncRNA signature (KTN1-AS1, LINC00460, and RP5-894A10.6) derived from the Atlas of ncRNA in cancer database could predict the survival of HNSCC patients, and a three-lncRNA signature (RP11-366H4.1.1, LINC00460, and AC093850.2) could predict overall survival and disease-free survival in patients with esophageal squamous cell carcinoma [25, 27]. Fang et al. also used the TCGA database and constructed a 13-lncRNA signature; however, it was noteworthy that the clinical utility of 13-lncRNA signature was limited due to too many biomarkers included [28]. In the present study, the data mining strategies were in accordance with studies previously reported by Sui et al. [13] and Zeng et al. [14]. The results showed that the six-lncRNA prognostic signature showed greater prognostic performance than the mRNA and miRNA signatures, which was a promising choice for clinical practice.

Among the six candidate lncRNAs, three have positive coefficients in the prognostic model and are associated with worse survival. Notably, HOTTIP showed the greatest prognostic value and was significantly correlated with poor prognosis (P<0.001). The lncRNA HOTTIP (HOXA transcript at the distal tip) located at the 5' end of the HOXA cluster, which is a key locus control element of HOXA genes, has been recently reported to be characterized by its key roles in carcinogenesis and progression of cancers. Aberrant expression of HOTTIP has been validated in various malignancies, including hepatocellular carcinoma [29], colorectal cancer [30], and gastric cancer [31]. Furthermore, upregulation of HOTTIP was reported to be significantly associated with advanced clinical stages, positive lymph node metastasis, drug resistance, and poor clinical outcome of cancer patients [32–34]. In the present study, we also analyzed HOTTIP expression pattern and its prognostic value in many other types of cancers. HOTTIP was shown to be downregulated in cervical cancer but upregulated in colon adenocarcinoma, rectum adenocarcinoma, and stomach adenocarcinoma. Moreover, HOTTIP showed prognostic value in lung squamous cell carcinoma and kidney renal clear cell carcinoma (Supplementary Figure 5). Apart from prognostic value, HOTTIP also exhibited potential diagnostic value in cancers. Zhao et al. found that exosomal HOTTIP demonstrated a high diagnostic capability in gastric cancer [35].

Many cytological and zoological experiments also validated HOTTIP's role in promoting cell proliferation, increasing cell migration and invasion, and inhibiting cell apoptosis [36–38]. In our study, upregulation of HOTTIP was shown to be related to clinical features, such as clinical stages and histological grade in HNSCC. Moreover, through functional assessment of potential target genes of HOTTIP and DEGs identified by HOTTIP expression level, it is suggested that HOTTIP might play important roles in tumorigenesis and progression of HNSCC. In addition, many studies have shown that HOTTIP could function as a ceRNA to promote the expression of oncogenic genes in renal cell carcinoma, lung cancer, and prostate cancer [37, 39, 40].

## 5. Conclusion

In summary, the construction of HNSCC-specific ceRNA network offers a novel insight into the competitive interactions between different types of RNAs in HNSCC, and it provides candidate prognostic biomarkers for HNSCC. More importantly, our study suggested that HOTTIP may play an important role in the tumorigenesis and progression in HNSCC, which might serve as a potential diagnostic, therapeutic, or prognostic biomarker in HNSCC.

## Data Availability

The data used to support the findings of this study are available from the corresponding author upon request.

## Supplementary Materials

Supplementary Materials
* Supplementary Figure 1. The differential expressions of RNAs in head and neck squamous cell carcinoma.* Volcano plots: (A) mRNAs; (B) lncRNAs; (C) miRNAs. Heatmaps: (D) mRNAs; (E) lncRNAs; (F) miRNAs. In the heatmaps, the X-axis represents samples and the Y-axis represents RNAs. Red denotes significant upregulation and green denotes significant downregulation (fold changes >2, adjusted P value <0.05).* Supplementary Figure 2. GO enrichment and KEGG pathway analysis of the differentially expressed genes*. Red denotes upregulated genes, green represents downregulated genes, and no color represents expanded genes.* Supplementary Figure 3. Kaplan–Meier survival curves of differentially expressed mRNAs* (A), lncRNAs (B–N), and miRNAs (O) involved in the HNSCC-specific ceRNA network.* Supplementary Figure 4. Time-dependent ROC curves of the RNA signatures derived from the ceRNA network for predicting 1-year, 3-year, 5-year, and 10-year survival of HNSCC patients in the TCGA dataset. Supplementary Figure 5. HOTTIP expression and its prognostic value in multiple cancers in TCGA database*. (A) HOTTIP expression profiling in multiple cancers in TCGA database. The green indicates downregulated and red indicates upregulated. (B) HOTTIP showed prognostic value in lung squamous cell carcinoma and kidney renal clear cell carcinoma. Supplementary Table 1. Differentially expressed mRNAs, lncRNAs, and miRNAs involved in the ceRNA network. Supplementary Table 2. Prognostic value of the differentially expressed mRNAs, lncRNAs, and miRNAs involved in the ceRNA network by univariate Cox regression analysis. Supplementary Table 3. Establishment of multifactor prognostic model by multivariate Cox analysis based on mRNAs, lncRNAs, and miRNAs expression involved in the ceRNA network. Supplementary File 1. differentially expressed mRNAs.xlsx. Supplementary File 2. differentially expressed lncRNAs.xlsx. Supplementary File 3. differentially expressed miRNAs.xlsx. Supplementary File 4. lncRNA_miRNA pair.xlsx.
