# Supplementary material for: HOTTIP Functions as a Key Candidate Biomarker in Head and Neck Squamous Cell Carcinoma by Integrated Bioinformatic Analysis
Source: Biomed Res Int. 2019 Mar 26;2019:5450617. doi: 10.1155/2019/5450617 (PMC6457310; doi:10.1155/2019/5450617)
Supplement: Supplementary Materials — Supplementary Figure 1. The differential expressions of RNAs in head and neck squamous cell carcinoma. Volcano plots: (A) mRNAs; (B) lncRNAs; (C) miRNAs. Heatmaps: (D) mRNAs; (E) lncRNAs; (F) miRNAs. In the heatmaps, the X-axis represents samples and the Y-axis represents RNAs. Red denotes significant upregulation and green denotes significant downregulation (fold changes >2, adjusted P value <0.05). Supplementary Figure 2. GO enrichment and KEGG pathway analysis of the differentially expressed genes. Red denotes upregulated genes, green represents downregulated genes, and no color represents expanded genes. Supplementary Figure 3. Kaplan–Meier survival curves of differentially expressed mRNAs (A), lncRNAs (B–N), and miRNAs (O) involved in the HNSCC-specific ceRNA network. Supplementary Figure 4. Time-dependent ROC curves of the RNA signatures derived from the ceRNA network for predicting 1-year, 3-year, 5-year, and 10-year survival of HNSCC patients in the TCGA dataset. Supplementary Figure 5. HOTTIP expression and its prognostic value in multiple cancers in TCGA database. (A) HOTTIP expression profiling in multiple cancers in TCGA database. The green indicates downregulated and red indicates upregulated. (B) HOTTIP showed prognostic value in lung squamous cell carcinoma and kidney renal clear cell carcinoma. Supplementary Table 1. Differentially expressed mRNAs, lncRNAs, and miRNAs involved in the ceRNA network. Supplementary Table 2. Prognostic value of the differentially expressed mRNAs, lncRNAs, and miRNAs involved in the ceRNA network by univariate Cox regression analysis. Supplementary Table 3. Establishment of multifactor prognostic model by multivariate Cox analysis based on mRNAs, lncRNAs, and miRNAs expression involved in the ceRNA network. Supplementary File 1. differentially expressed mRNAs.xlsx. Supplementary File 2. differentially expressed lncRNAs.xlsx. Supplementary File 3. differentially expressed miRNAs.xlsx. Supplementary File 4. lncRNA_miRNA pai [file 5450617.f1.zip › New folder/Supplementary Tables.docx]

**Supplementary Table 1. Differentially expressed mRNAs, lncRNAs and miRNAs involved in the ceRNA network.**

| **RNA** | **Down-regulated** | **Up-regulated** |
| --- | --- | --- |
| **mRNA** | SAMD5, CHRDL1, KLHL40, TGFBR3,  ALOX12, TPM2, SH3BGRL2, TMEM100,  GPD1L, GREM2 | POU3F2, HOXC8, NPTX1, IL11, STC2, HOXA10 |
| **lncRNA** | PART1, C20orf166-AS1, AL357153.1, LINC00314, AC004832.1, MUC19, ADARB2-AS1, DNMBP-AS1, SRGAP3-AS2, HCG22, SACS-AS1, LINC00443, FAM41C, AL161645.1, IDI2-AS1, ZFY-AS1, LINC00284, LINC00330, ATP13A5-AS1, AP000438.1, ADAMTS9-AS1, ADAMTS9-AS2, AL132855.1, ALDH1L1-AS2, LINC00492, RMST | NAALADL2-AS2, RRM1-AS1, ANO1-AS2, AC006372.1, POU6F2-AS2, LSAMP-AS1, AL139147.1, AC009093.1, LINC00461, ABCA9-AS1, WT1-AS, HOTTIP, MIAT, ERVH48-1, C8orf49,  TSPEAR-AS1, AL360004.1, LINC00355, AL163952.1, LINC00491, LINC00524, ERVMER61-1, LINC00486, LINC00487, LINC00221, MIR137HG, LINC00052, NOVA1-AS1, AL356056.1, LINC00525, AC078778.1, AL162427.1, SFTA1P, LINC00460, AL139002.1, AP002478.1, AC112721.1, AL035696.1， AP000525.1, HOTAIR， LINC00452， LINC00452, DLX6-AS1, TM4SF19-AS1,AC128709.1,  LINC00520 |
| **miRNA** | hsa-mir-301b  hsa-mir-31  hsa-mir-503  hsa-mir-210 | hsa-mir-211  hsa-mir-206  hsa-mir-195  hsa-mir-375 |

**Abbreviations:** HR: hazard ratio; CI: confidence interval.

**Supplementary Table 2. Prognostic value of the differentially expressed mRNAs, lncRNAs and miRNAs involved in the ceRNA network by univariate cox regression analysis.**

| **RNA** | **HR(95%CI)** | **z** | ***P* value** |
| --- | --- | --- | --- |
| **mRNA** |  |  |  |
| STC2 | 1.236 (1,120 - 1.364) | 4.213 | **<0.001 ***** |
| HOXC8 | 1.111 (1.024 - 1.204) | 2.536 | **0.011 *** |
| TGFBR3 | 0.889 (0.806 - 0.981) | -2.350 | **0.019 *** |
| **lncRNA** |  |  |  |
| LINC00460 | 1.120 (1.055 - 1.189) | 3.716 | **<0.001 ***** |
| HOTTIP | 1.183 (1.082 - 1.293) | 3.696 | **<0.001 ***** |
| AP002478.1 | 1.159 (1.068 - 1.258) | 3.523 | **<0.001 ***** |
| AL163952.1 | 1.103 (1.034 - 1.177) | 2.983 | **0.003 **** |
| MIAT | 0.882 (0.811 - 0.959) | -2.939 | **0.003 **** |
| LINC00052 | 1.096 (1.030 - 1.166) | 2.891 | **0.004 **** |
| ABCA9-AS1 | 1.104 (1.029 - 1.185) | 2.759 | **0.006 **** |
| ZFY-AS1 | 0.881 (0.803 - 0.966) | -2.690 | **0.007 **** |
| ANO1-AS2 | 1.095 (1.022 - 1.173) | 2.569 | **0.010 **** |
| HOTAIR | 1.106 (1.023 - 1.195) | 2.531 | **0.011 *** |
| AL161645.1 | 0.910 (0.840 - 0.985) | -2.344 | **0.019 *** |
| MIR137HG | 1.080 (1.012 - 1.153) | 2.309 | **0.021 *** |
| MUC19 | 0.901 (0.820 - 0.990) | -2.165 | **0.030 *** |
| SFTA1P | 1.068 (1.004 - 1.137) | 2.072 | **0.038 *** |
| LINC00520 | 1.071 (1.004 - 1.144) | 2.065 | **0.039 *** |
| **miRNA** |  |  |  |
| hsa-mir-206 | 1.037(1.004 - 1.070) | 2.207 | **0.027 *** |

**Abbreviations:** HR: hazard ratio; CI: confidence interval.

**Supplementary Table 3. Establishment of multi-factor prognostic model by multivariate cox analysis based on mRNAs, lncRNAs and miRNAs expression involved in the ceRNA network.**

| **RNA** | **HR(95%CI)** | **z** | ***P* value** |
| --- | --- | --- | --- |
| **mRNA** |  |  |  |
| TGFBR3 | 0.914 (0.829 - 1.008) | -1.80 | 0.072 |
| STC2 | 1.194 (1.076 - 1.325) | 3.35 | **<0.001 ***** |
| HOXC8 | 1.064 (0.977 - 1.158) | 1.42 | 0.155 |
| Likelihood ratio test = 22.8, ***p* value** = 4.44e-05 **<0.001 ***** | | | |
| **lncRNA** |  |  |  |
| LINC00052 | 1.065 (0.999 - 1.135) | 1.940 | 0.052 |
| ZFY-AS1 | 0.890 (0.811 - 0.976) | -2.460 | **0.014 *** |
| ABCA9-AS1 | 1.087 (1.009 - 1.170) | 2.210 | **0.027 *** |
| MIAT | 0.908 (0.834 - 0.989) | -2.220 | **0.026 *** |
| HOTTIP | 1.170 (1.067 - 1.283) | 3.330 | **<0.001 ***** |
| LINC00460 | 1.072 (1.006 - 1.143) | 2.130 | **0.033 *** |
| Likelihood ratio test = 46.1, *p* value = 2.82e-08**<0.001 ***** | | | |
| **miRNA** |  |  |  |
| hsa-mir-206 | 1.037 (1.004 - 1.070) | 2.210 | **0.027 *** |
| Likelihood ratio test = 4.85, *p* value = **0.028 *** | | | |

**Abbreviations:** HR: hazard ratio; CI: confidence interval.
